# Uncovering the biases: why the claimed mask–excess mortality link fails to hold

**DOI:** 10.1016/j.lana.2025.101360

**Published:** 2025-12-29

**Authors:** Thiago Cerqueira-Silva, Felipe Argolo, Gabriel Gonçalves da Costa, Felipe Nogueira Barbara, Pedro Hallal, Bruno Gualano

**Affiliations:** a Medical Statistics Department, London School of Hygiene and Tropical Medicine, London, UK; b Departamento de Psiquiatria, Faculdade de Medicina, Universidade de São Paulo, Sao Paulo, Brazil; c Instituto de Bioquímica Médica Leopoldo de Meis, Universidade Federal do Rio de Janeiro, Rio de Janeiro, Brazil; d Independent Researcher, Former PGCM, Universidade do Estado do Rio de Janeiro, Rio de Janeiro, Brazil; e Department of Health and Kinesiology, University of Illinois Urbana-Champaign, Champaign, IL, USA; f Centro de Medicina do Estilo de Vida, Faculdade de Medicina, Universidade de São Paulo, Sao Paulo, Brazil

The relationship between mask usage and excess mortality during the COVID-19 pandemic remains a subject of ongoing debate. A recent article by Tausk and Spira, “*Does mask usage correlate with excess mortality? Findings from 24 European countries*,”^1^ reported a positive correlation between mask use and excess mortality in 2020 and 2021. This finding attracted considerable attention, particularly in Brazil, where the authors are based. The study's ecological design, which is susceptible to bias when assessing individual-level exposures (such as mask use) compared to aggregate exposures (such as citywide pollution), has prompted widespread discussion on social media platforms. Brazil's former President Jair Bolsonaro publicly stated that the University of São Paulo — the largest university in Latin America and the authors' affiliation — “owed him an apology”.^2^ His reaction was consistent with a pattern of denialist discourse during the pandemic, for which he was widely criticised by scholars, health professionals, and the media.^3^ For Bolsonaro and supporters, the study served as “proof” that masks not only failed to prevent COVID-19 but also increased deaths.

In our view, the authors' approach relies on strong — and flawed — assumptions. A key limitation is their decision to consider only average mask usage across 2020–2021, overlooking how it changed over time. During the pandemic, mask use generally increased alongside non-pharmaceutical interventions (NPIs), often implemented in response to rising deaths. In such cases, any observed correlation between high mask usage and high mortality may simply indicate more severe local outbreaks rather than masks causing worse outcomes. We plotted the moving average of mask usage across all 24 countries included in the original study (Fig. 1). Even in countries with average use above 50%, some weeks fell below 25%. Similarly, in countries averaging 35% or lower, usage sometimes exceeded 50%. To further contextualise the findings, we included data from Brazil (Fig. 1d) showing the excess mortality (monthly) and mask usage (30-day moving average). The figure suggests that an increase in mask adoption typically occurs after peaks in mortality, indicating a reactive response to worsening outbreaks rather than a causal effect, contradicting Tausk and Spira's conclusions.

**Fig. 1:**
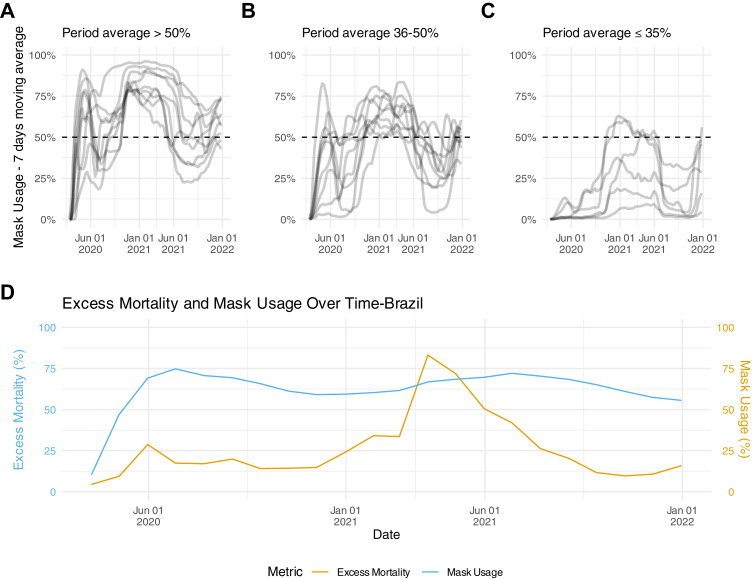
**Mask usage percentage by country from March 2020 to December 2021, with each line representing one country. (A**–**C)** 7-day moving average; **(D)** 30-day moving average. **(A)** Nine countries have an average mask usage rate higher than 50%. **(B)** Nine countries have an average mask usage between 36% and 50%. **(C)** Six countries have an average mask usage of 35% or lower. **(D)** Data from Brazil on monthly excess mortality and mask usage.

To illustrate these points, we re-analysed data from the same source (the Institute for Health Metrics and Evaluation, IHME). Unlike the original study, we used weekly moving averages instead of the entire-period average mask-usage data and accounted for country-level differences using two-way fixed effects (full description of methodology in Appendix pp 2 and 3). We evaluated the association between mask and weekly excess mortality and the cumulative weekly excess mortality one to four weeks later. Our results did not support a positive correlation between mask use and excess mortality. Instead, we found a small statistically significant negative association for cumulative excess mortality in all lags evaluated, ranging from −0.067 to −0.071 (Appendix p 3). In the analysis of weekly excess mortality, we found no statistically significant association in all lags evaluated, ranging from 0.013 to 0.052 (Appendix p 3). This divergence between cumulative and weekly estimates warrants clarification. Cumulative excess mortality aggregates information over time and is therefore less sensitive to short-term variability and weekly noise, but it may introduce distinct biases by smoothing over heterogeneous epidemic phases. In contrast, weekly excess mortality provides a more temporally granular indicator, though its higher variability reduces statistical power. The absence of an association in the weekly analysis may thus reflect lower precision, while the cumulative result, although statistically significant, should be interpreted cautiously given its susceptibility to aggregation bias.

We also assessed the association between NPIs (using the Oxford Stringency Index,^4^ which ranges from 0 to 100) and subsequent mask usage. Our models showed a statistically significant positive association between NPIs and mask usage in all lags evaluated, ranging from 0.286 to 0.324 (Appendix p 3), meaning each one-point increase in the Stringency Index was associated with a 0.28–0.32% rise in mask use in the following weeks. Taken together, these findings suggest that higher mask usage is more likely a response to worsening COVID-19 conditions than a cause of increased mortality. While our analytical approach addressed several methodological limitations of the original study, it remains ecological in nature and is therefore subject to the same inferential constraints. Our aim was not to provide definitive evidence on the relationship between mask use and mortality; such conclusions would require individual-level causal designs. Rather, we sought to show the fragility of the original ecological approach, whose conclusions shift substantially under different model specifications and temporal resolutions.

Following criticism, Tausk and Spira recently issued an extensive correction,^5^ primarily to acknowledge that their observational data cannot support causal claims. Although the overt causal language has been removed, the authors continue to highlight a significant ecological association between higher mask usage and “unintended adverse effects” (i.e., higher mortality). In addition to the aforementioned methodological biases, this interpretation lacks clinical and biological plausibility. Substantial evidence shows that masks, especially high-grade masks used correctly, are effective barriers to viral transmission or, at worst, do not increase infections or mortality.^6^, ^7^, ^8^, ^9^, ^10^, ^11^

Our re-analysis indicates that the reported association between mask usage and excess mortality presented by Tausk and Spira remains misleading, even after their correction. The findings are undermined by substantial methodological flaws, a lack of biological plausibility, and both interpretive and ecological biases. Given the risk of misinforming scientific understanding and public health policy, the study should not be used to inform scientific or public health debates in its current form.

## Contributors

TCS–Project administration, Conceptualization, Methodology, Visualization, Investigation, Supervision, Data curation, Formal analysis, Writing—original draft.

FA–Visualization, Investigation, Conceptualization, Supervision, Data curation, Formal analysis, Validation, Writing—original draft, Writing–review.

GGC–Visualization, Investigation, Conceptualization, Data curation, Formal analysis, Validation, Writing—original draft, Writing–review.

FNB–Investigation, Conceptualization, Data curation, Writing—original draft, Writing—review.

PH–Visualization, Supervision, Writing—original draft, Writing—review & editing.

BG–Visualization, Supervision, Writing—original draft, Writing—review & editing.

## Data sharing statement

The complete analysis code and data are available at: https://github.com/csthiago/reanalysis_mask.

## Declaration of interests

None to declare.
