# Supplementary material for: Uncovering the biases: why the claimed mask–excess mortality link fails to hold
Source: Lancet Reg Health Am. 2025 Dec 29;55:101360. doi: 10.1016/j.lana.2025.101360 (PMC12803879; doi:10.1016/j.lana.2025.101360)
Supplement: Appendix [file mmc1.docx]

**Supplementary appendix**

Supplement to: Cerqueira-Silva T, et al. Uncovering the Biases: Why the Claimed Mask–Excess Mortality Link Fails to Hold. *Lancet Regional Health – Americas*

Table of Contents

[Additional Methods: 2](#_Toc216257028)

[Study Design 2](#_Toc216257029)

[Data Sources 2](#_Toc216257030)

[Descriptive Analyses 2](#_Toc216257031)

[Study Population and period 2](#_Toc216257032)

[Exposure Variable 2](#_Toc216257033)

[Outcome Variables 2](#_Toc216257034)

[Statistical Analysis 2](#_Toc216257035)

[Data and Code Availability 3](#_Toc216257036)

[Additional Results 3](#_Toc216257037)

# Additional Methods:

## Study Design

We conducted an ecological analysis using country-level longitudinal data to examine the association between mask usage and excess mortality during the COVID-19 pandemic, evaluating the same set of countries evaluated by Tausk and Spira.^1^ In our reanalysis, we utilised weekly moving averages to account for temporal variation in both exposure and outcome measures, while Tausk and Spira used period-averaged mask usage across 2020–2021.

## Data Sources

Data were obtained from two primary sources:

**Institute for Health Metrics and Evaluation (IHME) (**[**https://www.healthdata.org/research-analysis/health-topics/covid-research-library**](https://www.healthdata.org/research-analysis/health-topics/covid-research-library)**), daily** mask usage between 2020 and 2021;

**Our World in Data (OWID) (**[**https://github.com/owid/covid-19-data/tree/master**](https://github.com/owid/covid-19-data/tree/master)**), in** COVID-19 epidemiological indicators and excess mortality estimates (weekly and cumulative)

## Descriptive Analyses

We classified the countries used in the analysis by Tausk and Spira by mean mask usage over the study period (2020-2021) into three categories: high (>50%), moderate (36–50%), and low (≤35%). We then plotted the weekly mean mask usage by country in each category to examine heterogeneity in mask usage over time.

A separate descriptive analysis for Brazil was conducted using a 30-day moving average for mask usage to examine the temporal relationship between excess mortality peaks and subsequent changes in mask adoption.

## Study Population and period

The analysis included the same 24 European countries examined in the original study by Tausk and Spira.^1^ We used data from 01 January 2020 to 02 January 2022 (the last ISO week of 2021).

## Exposure Variable

The primary exposure was mask usage, defined as the 7-day moving average of the mean mask use prevalence in percentage. We used the value of the moving average in each Sunday for the weekly analysis.

## Outcome Variables

We used two outcome measures (**Weekly excess mortality and cumulative excess mortality)**, evaluated 1 to 4 weeks later, as it is not expected immediate effect of any policy measure on the excess mortality.

## Statistical Analysis

The primary analysis employed two-way fixed effects regression models. Fixed effects were specified for both country (location) and time (week-year) to control for time-invariant country-level confounders (e.g., healthcare infrastructure, population demographics) and common temporal trends affecting all countries (e.g., emergence of new variants, global public health guidance). We used a two-way cluster-robust standard errors to account for potential correlation of errors within countries over time and across countries within time periods.

## Data and Code Availability

The complete analysis code and data are available at: <https://github.com/csthiago/reanalysis_mask>

# Additional Results

Supplementary Table 1: Effect of mask usage on weekly excess mortality (%) and cumulative excess mortality (%) by different lags.

| **Lag** | **Weekly excess mortality β (95% CI)** | **Cumulative excess mortality β (95% CI)** |
| --- | --- | --- |
| 1 week | 0.0524 [-0.1008; 0.2057] | -0.0674 [-0.1270; -0.0077] |
| 2 weeks | 0.0426 [-0.1015; 0.1867] | -0.0688 [-0.1269; -0.0107] |
| 3 weeks | 0.0269 [-0.1107; 0.1644] | -0.0702 [-0.1263; -0.0140] |
| 4 weeks | 0.0134 [-0.1207; 0.1476] | -0.0713 [-0.1253; -0.0172] |

Supplementary Table 2: Effect of Oxford stringency on mask usage by different lags.

| **Lag** | **β (95% CI)** |
| --- | --- |
| 1 week | 0.3233 [0.1396; 0.5069] |
| 2 weeks | 0.3240 [0.1540; 0.4940] |
| 3 weeks | 0.3105 [0.1543; 0.4667] |
| 4 weeks | 0.2865 [0.1364; 0.4366] |

Reference:

1 Tausk DV, Spira B. Does mask usage correlate with excess mortality? Findings from 24 European countries. *BMC Public Health* 2025; **25**: 913.
